# Implementing Personalized Medicine in COVID-19 in Andalusia: An Opportunity to Transform the Healthcare System

**DOI:** 10.3390/jpm11060475

**Published:** 2021-05-26

**Authors:** Joaquín Dopazo, Douglas Maya-Miles, Federico García, Nicola Lorusso, Miguel Ángel Calleja, María Jesús Pareja, José López-Miranda, Jesús Rodríguez-Baño, Javier Padillo, Isaac Túnez, Manuel Romero-Gómez

**Affiliations:** 1 GT MP Covid-19. SGIDIS, Consejería de Salud y Familias, Junta de Andalucía, Spain; joaquin.dopazo@juntadeandalucia.es (J.D.); fegarcia@ugr.es (F.G.); nicola.lorusso.sspa@juntadeandalucia.es (N.L.); mangel.calleja.sspa@juntadeandalucia.es (M.Á.C.); maria.pareja.sspa@juntadeandalucia.es (M.J.P.); md1lomij@uco.es (J.L.-M.); jesusrb@us.es (J.R.-B.); javierpadilloruiz@gmail.com (J.P.); 2 Área de Bioinformática, Fundación progreso y Salud, Junta de Andalucía, 41013 Sevilla, Spain; 3 Centro de Investigación Biomédica en Red de Enfermedades Raras (CIBERER), 28029 Madrid, Spain; 4 Instituto de Biomedicina de Sevilla (HUVR/HUVM/CSIC/US), 41013 Sevilla, Spain; dmaya-ibis@us.es; 5 Centro de Investigación Biomédica en Red de Enfermedades Hepáticas y Digestivas (CIBEREHD), 28029 Madrid, Spain; 6 Servicio de Microbiología, Hospital Universitario San Cecilio, 18016 Granada, Spain; 7 Instituto de Investigación Biosanitaria IBS, 18012 Granada, Spain; 8 Dirección General de Salud Pública, Consejería de Salud y Familias, Junta de Andalucía, Spain; 9 Servicio de Farmacia, Hospital Universitario Virgen Macarena, 41009 Sevilla, Spain; 10 Hospital Universitario de Valme, 41014 Sevilla, Spain; 11 Servicio de Medicina Interna, Hospital Universitario Reina Sofía, 14004 Córdoba, Spain; 12 Instituto Maimónides de Investigación Biomédica de Córdoba (IMIBIC), 14004 Córdoba, Spain; 13 Departamento de Ciencias Médicas y Quirúrgicas, Universidad de Córdoba, 14071 Córdoba, Spain; 14 Unidad Clínica de Enfermedades Infecciosas y Microbiología, Hospital Universitario Virgen Macarena, 41009 Sevilla, Spain; 15 Departamento de Medicina, Universidad de Sevilla, 41009 Sevilla, Spain; 16 Departamento de Cirugía, Universidad de Sevilla, 41009 Sevilla, Spain; 17 Servicio de Cirugía General y Digestiva, Hospital Universitario Virgen del Rocío, 41013 Sevilla, Spain; 18 Departamento de Bioquímica, Universidad de Córdoba, 14071 Córdoba, Spain; 19 Secretaría General de Investigación, Desarrollo e Innovación en Salud, Consejería de Salud y Familias de la Junta de Andalucía, 41020 Sevilla, Spain; 20 Servicio de Aparato Digestivo, Hospital Universitario Virgen del Rocío, 41013 Sevilla, Spain

**Keywords:** personalized medicine, precision medicine, Covid-19, SARS CoV2, epidemiology, host genetics, viral genome

## Abstract

The COVID-19 pandemic represents an unprecedented opportunity to exploit the advantages of personalized medicine for the prevention, diagnosis, treatment, surveillance and management of a new challenge in public health. COVID-19 infection is highly variable, ranging from asymptomatic infections to severe, life-threatening manifestations. Personalized medicine can play a key role in elucidating individual susceptibility to the infection as well as inter-individual variability in clinical course, prognosis and response to treatment. Integrating personalized medicine into clinical practice can also transform health care by enabling the design of preventive and therapeutic strategies tailored to individual profiles, improving the detection of outbreaks or defining transmission patterns at an increasingly local level. SARS-CoV2 genome sequencing, together with the assessment of specific patient genetic variants, will support clinical decision-makers and ultimately better ways to fight this disease. Additionally, it would facilitate a better stratification and selection of patients for clinical trials, thus increasing the likelihood of obtaining positive results. Lastly, defining a national strategy to implement in clinical practice all available tools of personalized medicine in COVID-19 could be challenging but linked to a positive transformation of the health care system. In this review, we provide an update of the achievements, promises, and challenges of personalized medicine in the fight against COVID-19 from susceptibility to natural history and response to therapy, as well as from surveillance to control measures and vaccination. We also discuss strategies to facilitate the adoption of this new paradigm for medical and public health measures during and after the pandemic in health care systems.

## 1. Introduction

Policymakers, health care leaders and physicians should improve our response to the SARS-CoV-2 pandemic by promoting interdisciplinary collaboration and moving the main research and innovation milestones to clinical practice. The management of this pandemic has been a great challenge addressing health care delivery in stressful conditions due to inadequate capacity, supply shortages, redesigning care, being more transversal and focused on the patient and the virus, thinking in a few days on open intensive care units distributed throughout the campus and managed by several specialties. Innovation and research on COVID-19 required an adaptive process to be translated to clinicians as fast as possible when usefulness was supported in evidence-based medicine. Complex adaptive systems that operate in unpredictable environments should be replaced. Personalized medicine implementation in clinical practice requires a multidisciplinary approach putting together people working on genome sequences, bioinformatics, geneticists or microbiologists and physicians in charge of the patients as well as the management of big-data (BD). A well-defined circuit is mandatory together with a multidisciplinary group able to meet frequently to solve complex clinical situations as severe COVID-19. Precision medicine and beyond, P4 medicine, including predictive, personalized, preventive and participatory medicine, could find the correct scenario in this pandemic. The Andalusian Regional Government allowed us to put together a large population-based health database together with human genomics and viral sequencing to be addressed by Artificial Intelligence (AI) methods to develop robust algorithms able to predict not just the natural history and progression of the disease but also antiviral therapy response [1] and immune response to vaccination [2].

## 2. Impact of Human Genome on COVID-19

The clinical course in patients with COVID-19 has been reported as highly heterogeneous. While most people will experience a mild or asymptomatic course, some others may develop progressive and life-threatening bilateral pneumonia and acute respiratory distress syndrome (ARDS). Identifying which patients are at risk to progress to a severe form could reduce the burden of COVID-19, which is currently overloading many health care systems around the world. Factors contributing to disease severity include anthropometric factors (e.g., age, gender, BMI), comorbidities (e.g., hypertension, diabetes) and unhealthy habits (e.g., smoking) [3]. Host genetics studies in COVID-19 have reported genomic variations associated with disease severity in chromosomes 1 (1q22.1), 2 (2p21.1), 3 (3p21.1–3), 6 (6p21.1), 8 (8q24.13), 9 (9q34.1–2), 12 (12q24.1–2), 17 (17q21.3), 19 (19p13.1–3) and 21 (21q21–q22) as well as in specific loci that have been manually selected [4,5,6,7,8]. The COVID Host Genetics Initiative has set up one of the largest communities that are currently generating, sharing and analyzing data to learn the genetic determinants of COVID-19 susceptibility, severity and outcomes. This initiative, which has currently released its fifth data freeze, including data from 46 studies across 19 countries worldwide and analysis looking for genetic determinants of severity (+6000 death or intubated patients vs +1.4 M controls), hospitalization (+13,000 hospitalized patients vs +2 M controls) and infection (≈ 50 K infected patients vs +1.7 M controls), is currently setting up a platform in which researchers will be able to explore the genetic variations that have a deeper impact in SARS CoV-2 infection and severity [9]. An overview of some of the strongest genetic associations described so far for COVID-19 infection and severity, along with some potential gene candidates, is shown in Table 1. Chromosome 3 genetic variation in the 3p21 locus is the genetic variant that has shown the strongest association in terms of reproducibility to both COVID-19 infection and severity. This region, which is thought to be present in around 30% of people in South Asia and 8% in Europe, has been shown to increase between 1.5- and 2-times approximately an infected person’s odds of developing severe COVID-19 [4,7,10]. Carriers of rs10490770, an SNP strongly linked to this chromosome region have increased the odds of several COVID-19 complications, including severe respiratory failure (odds ratio [OR] 2·0, 95%CI 1·6–2·6), venous thromboembolism (OR 1·7, 95%CI 1·2–2·4), and hepatic injury (OR 1·6, 95%CI 1·2–2·0) and higher odds of death or severe respiratory failure, which are especially relevant in patients ≤60 years (OR 2.6, 95%CI 1.8–3.9) compared to those >60 years (OR 1.5 (95%CI 1.3–1.9, interaction *p*-value = 0·04) [11]. rs11385942, another SNP strongly associated with this genetic variation, has shown no association to biomarkers of systemic inflammation, including the C-reactive protein, ferritin, IL-6 and circulating neutrophils and lymphocytes but has been recently associated to increased amounts of the enhanced complement activation, both with C5a and terminal complement complex [12,13]. The 3p21.31 locus contains 17 known protein-coding genes, including SLC6A20, LZTFL1, CCR9, FYCO1, CXCR6, XCR1, CCR1, CCR3, CCR2 and CCR5. None of them seems to have a straightforward connection to SARS-CoV-2 infection or severity to date, besides maybe SLC6A20, which is a transporter regulated by the main SARS-CoV-2 receptor ACE2. However, there are some indirect connections that might be worth highlighting. CCR2 encodes a C-C type chemokine receptor for a chemokine (CCL2) that mediates monocyte chemotaxis. Its expression has recently shown a strong association with the 3p21.31 severe COVID-19-risk variant in certain CD4+ memory T cell subsets and classical monocytes. Patients with severe COVID-19 illness have increased CCR2 expression in circulating monocytes as well as very high levels of CCR2 ligand (CCL2) in bronchoalveolar lavage fluid, leading to the hypothesis that excessive recruitment of CCR2-expressing monocytes may drive pathogenic lung inflammation in COVID-19 [14,15]. LZTFL1/BBS17 is a member of the Bardet-Biedl syndrome (BBS) and encodes a protein involved in protein trafficking to primary cilia. Mutations in LZTFL1 have been reported in human BBS patients, which develop a wide range of pathologies, including obesity, which is so far one of the comorbidities with a stronger link to both COVID-19 infection and severity. It has been recently shown that the deletion of LZTFL1 can cause pleiotropic defects in mice, including obesity. Interestingly, this work links obesity to the expression of LZTFL1 in the brain and demonstrates that the loss of this protein specifically in the brain can lead to leptin resistance [16]. SARS-CoV-2 has been shown to be able to infect the epithelial cells of the gastrointestinal glands of the stomach, duodenum and rectum of COVID-19 patients. The continuous positive detection of SARS-CoV-2 viral RNA in stools suggests that viral particles can be secreted by gastrointestinal cells infected with the virus, and two recent works have proven the ability of this virus to infect human and bat enterocytes in vitro [17,18,19]. CCR9 is a small intestinal chemokine homing receptor normally found on most mucosal T cells in the gastrointestinal tract, and that has been linked to celiac disease [20] It has been recently shown that the CCR9-CCL25 axis in mice promotes the development of a Th1 population with features of TRM cell that regulates the local immune environment and that CCR9 can exert a protective response against infections in the gastrointestinal tract [21]. CCR9 expression has also been observed in inflammatory cells that are recruited to the lungs and in peripheral blood eosinophils of asthmatic subjects and can be upregulated by stimulation with proinflammatory mediators in human eosinophils-derived cell lines [22]. FYCO1 plays a role in microtubule plus end-directed transport of autophagic vesicles through interactions with the small GTPase Rab7. Although the molecular mechanism of SARS-CoV2 virus infection and spread in the body is not yet disclosed, studies on other beta-coronaviruses show that, upon cell infection, these viruses inhibit macroautophagy/autophagy flux and cause the accumulation of autophagosomes. RNA viruses such as HBV and HCV also modify the autophagy machinery to favor viral replication, translation and propagation. [23] Experiments performed in hepatocyte cell lines have shown that HCV infection causes inhibition of ras-related protein Rab-7 (Rab7)-dependent endosome–lysosome fusion and promotes the cleavage of the Rab7 adaptor protein RILP (Rab interacting lysosomal protein). This cleavage allows changing the location of this protein to the cell periphery, promoting the export of viral particles outside the cell [24]. XCR1 is a chemokine receptor for XCL1 (Lymphotactin or Lptn). This chemokine is produced predominantly by NK and CD8+ T cells and plays a key role in the tissue-specific recruitment of T lymphocytes [25]. Nasal co-administration of XCL1 and a protein antigen enhances antigen-specific antibody responses both in blood plasma and in mucosal secretions. Lptn as adjuvant-induced antigen-specific CD4+ Th1- and Th2- type cells and IgG1 > IgG2a = IgG2b = IgG3 antibody subclasses [26]. Viral macrophage inflammatory protein-II, a viral protein that inhibits C class chemokines, has been shown to be a potent antagonist able to block the signaling of the XCR1 receptor [27], and the TAT protein of HIV can strongly increase the expression of XCL1 in several cell types [28].

## 3. Role of Viral Genetic Variants in Covid-19

Coronaviruses (CoVs) are positive ssRNA viruses, non-fragmented, 26–32 kb belonging to the coronaviridae family. At least four types have been described (α, β, γ and δ). Alpha and beta are pathogenic to mammalians, including humans. Until now, they have been associated with respiratory diseases—α: HCoV-229E and HCoV-NL63 and β: HCoV-OC43, HCoV-HKU1, SARS-CoV-1, MERS-CoV and SARS-CoV-2. The virus did infect and replicate in the cell-expressing ACE-2 receptors. Infection is diagnosed by RT-PCR detecting at least two of these four genes (envelope, spike, nucleocapsid or RNA-dependent RNA polymerase) [29]. The enormous international effort in SARS-CoV-2 sequencing and the subsequent data sharing in sequence databases, along with the popularization of the interactive epidemiological map viewer Nexstrain [30], has uncovered a huge variability spectrum in the viral sequences reported, which are estimated to accumulate nucleotide mutations at a rate of about 1–2 mutations per month [31]. As new sequences cumulated, an increasing number of studies started to describe mutations with an apparent impact on increased infectivity of the virus, such as the well-known D614G variant in the spike protein, or even on increased mortality [32]. Additionally, mutations in the spike have been associated with mABs evasion, which implies a potential risk for vaccine effectivity [33]. Beyond the spike protein, mutations in other proteins, such as the RNA-dependent-RNA polymerase, can reduce the copy fidelity that could result in resistance to antiviral treatments [34] On the other hand, mutations present at the receptor-binding site of the spike protein apparently cause reduced infectivity [33]. ORF8 deletion has also been associated with a milder clinical infection and less post-infection inflammation [35]. Actually, it has been suggested that superspreading events seem to be driving the SARS-CoV-2 pandemic evolution [36]. Moreover, sometimes a more drastic evolutionary event can occur, such as the recently described transmission between humans and mink, and back to humans [37], which triggered a preventive systematic mink slaughter in Denmark because of the presence of variants in the spike protein that might compromise the effectivity of a vaccine [38]. Another case of a new SARS-CoV-2 variant with an unexpectedly large number of mutations in several proteins is the new lineage, B.1.1.7, described in the UK, which is apparently associated with higher transmission rates and mortality [39], or the more recent strains B.1.351 from South Africa and B.1.1.28.1 from Brazil.

The potential effect of many of these mutations has been questioned as speculative and often based on a small number of cases with no much clinical information associated, which casts serious doubts on their actual significance [40]. However, there is an obvious scenario where viral mutations are known to have a potential effect: resistance to antiviral treatments. A noticeable example is remdesivir, an antiviral agent developed against the Ebola virus with demonstrated in vitro activity against SARS-CoV-2. In vitro studies have linked some drug-resistance variants, mainly amino acid changes on RNA-dependent RNA-polymerase (corresponding to residues F480; V557; F480 + V553; F480 + V557), with reduced susceptibility to remdesivir (between 2.4- and 6-fold changes). In clinical cases, some emergent variants promoting drug resistance, mainly in the RNA-dependent RNA-polymerase region (D484Y), as a therapeutic target of the drug, have been reported in patients receiving remdesivir [41,42,43]. Indeed, new variants are continuously emerging every day and may affect drug binding sites and, consequently, may bear the potential to promote drug resistance or escape to current vaccines. Therefore, the benefit of combined genomic and epidemiological analysis for the investigation of health-care-associated COVID-19 seems apparent, as has recently been reported, enabling the detection of cryptic transmission events and identify opportunities for early interventions.

## 4. Genetic Epidemiology of COVID-19

In recent years, the European Center for Disease Prevention and Control (ECDC) has published several documents informing about both the roadmap for and state of the art on the current situation of the integration of microbiological data in Public Health surveillance and proposing the need to implement molecular typing and genomic sequencing data for outbreak surveillance and control [44,45]. The use of genomic surveillance and molecular typing in public health surveillance involves the availability of complementary information to the epidemiological survey and identification of contacts (allowing traceability of the transmission chain). It allows knowing if the cases belong almost unequivocally to the same exposition or transmission line. Using this technique, exposures to a common source can quickly and easily be identified, as demonstrated in the recently reported UK SARS-CoV-2 variant [46]. The creation of a regional geographic database allows to know which pathogens are endemic and which are imported and the identification of new clades, strains or variants that are imported. The analysis of genetic data with phylodynamic methods allows making inferences about the characteristics of the individuals involved in the transmission of the infection and about how contact patterns and the dynamics of risk behaviors affect the flow of transmission through a population [39]. Thus, epidemiological surveillance has to monitor for abrupt changes in rates of transmission or disease severity as part of a systematic process of identifying, response and assessing the impact of variants. A recent example has been the emergence and rapid spread of the above-mentioned new SARS-CoV-2 B.1.1.7 variant with multiple spike protein changes and mutations in other genomic regions associated with higher transmissibility [46].

Although retrospective incorporation has made it possible at the local level to secure the data that had been identified by epidemiologists through surveys when the virus sequencing of confirmed cases has to be carried out faster, the pathways of infection have been prospectively traced, and chains of transmission have been precisely identified, at the hospital level [44,45,47,48,49]. Technological advances in the classification of pathogens according to the genomic sequence propel us into a new era of massive availability of genomic data at increasingly reduced costs. This information applied to current surveillance systems allows the pathogen causing an infection to be more accurately discriminated, improves the detection of outbreaks and circumscribes the scope and impact of these outbreaks as it allows defining transmission patterns at an increasingly local level. SARS-CoV-2 sequencing has also proven to be useful for studying reinfection. The first case of SARS-CoV-2 reinfection was reported on 24 August 2020 in a Hong Kong citizen re-infected while travelling in Europe. A few other COVID-19 reinfections have been published or deposited in repositories; however, as diagnosing reinfection requires whole-genome sequencing strategies to evaluate the differences between the first and the second strain and samples from both episodes may not be available, it is suspected that reinfection may be a more frequent event than reported. 

Another important aspect of genomic surveillance of SARS-CoV-2 is related to vaccination. An effective vaccine should consider the natural variation of the pathogen in order to provide protection with coverage as extensive as possible^.^ The evolution of viruses by mutating epitopes to escape from different pressures has been demonstrated in vitro in the presence of monoclonal antibodies [50] and also in clinical trials [51]. Additionally, some cases of viruses that evade the immune response elicited by vaccines have been described [52,53]. As expected, SARS-CoV-2 can escape in vitro from neutralizing antibodies against the spike protein [54]. However, the recent report of the escape in vitro from a neutralizing COVID-19 convalescent plasma with only three mutations [55], is a serious warning that cannot be ignored and points to the convenience of surveillance that considers immune aspects. The use of immunogenomics, bioinformatics and systems biology helps to understand the basis of interindividual responses to vaccines, both in terms of acquired immunity and adverse effects. The application of these concepts opens the door to the possibility of quantifying and predicting the protective immune response induced by vaccines according to the genomic signature, both of the microorganism and of the vaccine recipient itself [56]. In fact, a bioinformatic approach has recently been described that can predict candidate targets for immune responses to SARS-CoV-2 [57], providing crucial information for understanding human immune responses to this virus and for evaluating vaccine candidates [58]. In fact, these predictions, based on Artificial Intelligence (AI) methodologies [59], allows understanding the individual responses of patients against the virus [60]. Thus, genomic surveillance and patient screening of risk variants need to be considered for personalized approaches to SARS-CoV-2 vaccination and to prevent possible future vaccine failures [61]. 

## 5. Data Science in Health Data Sheet from Large Populations: An Opportunity for COVID-19

Recent estimates suggest that, while more than 50 years were needed to duplicate all the medical knowledge by 1950, only 70 days were necessary for this increase by the end of 2020 [62], thus providing an idea of the real dimension of current clinical *BD* and their amazing growing pace. This increasing volume of data poses growing challenges for its management, but at the same time, offers an invaluable opportunity for discovery. In fact, the secondary use of stored clinical data is gaining importance progressively and provides a solid substrate for an increasing number of real-world evidence (RWE) studies [63]. Additionally, in parallel to the growth of clinical data repositories, the field of AI has recently experienced a remarkable development, particularly in the case of clinical applications [64]. The AI is starting to integrate into many aspects of medicine with the perspective of optimizing processes, diagnostic procedures and treatments, as well as helping to reduce medical errors [65].

It is worth noting that, despite the short time since the first COVID-19 outbreak, the activity in the development of applications for the retrospective analysis of electronic health records (EHR) by means of artificial intelligence (AI) applied to different aspects of the pandemic is remarkable [66]. With a rapidly growing amount of data available, predictors for different endpoints are being proposed based on different clinical data, that include a medical image [67], clinical text data [68] or, in general, clinical data contained in the EHRs [69]. One of the strengths of AI is its ability to “learn” how individual EHR variables (potential risk factors) can be used and combined among them to produce personalized risk predictions. While conventional approximations (e.g., Cox proportional hazards model) cannot properly combine heterogeneous data from different natures and often incomplete EHRs, modern AI techniques, based on supervised learning, can efficiently learn from such a complex variable dataset and generate risk predictors, as well as update their predictions as data evolve with time [70].

Beyond clinical data from EHRs, the abundance of genomic data in public databases, as well as international initiatives to rapidly increase the biological knowledge on the viral infection process, such as the COVID-19 disease map [71], is fostering innovative applications of AI in the field of drug repurposing [72]. It has recently been demonstrated that a combination of AI methods and mathematical models of the COVID-19 disease map^2^ has been able of predicting all targeted drugs for COVID-19 treatment currently in clinical trials [73], opening the door to a new era in which AI-based *in silico* studies will become mainstream. AI may also help in the design of new randomized trials by selecting the most appropriate subpopulations for testing specific drugs according to the best fitted a-priory hypothesis based on the mechanisms of action of the drugs.

## 6. Ethics, Data Science and Data Sharing in the Times of COVID-19

Sharing data and results arising from public research projects promotes scientific progress. This concept, widely accepted among research communities, funding bodies, and regulatory agencies, has acquired an unprecedented dimension during the COVID-19 pandemic. The scientific and medical communities have both put in enormous effort to promote data sharing as fast as possible in order to advance our knowledge during the pandemic and design more effective ways to fight it back. Huge efforts have been performed to identify biological and non-biological factors behind the enormous heterogeneity of COVID-19 outcomes and to design strategies able to improve the standard of care given to patients. Sharing clinical and genomic data can improve research efficiency, especially in the genetics field, where the number of samples required to achieve enough statistical power is high. The analysis and re-use of large datasets also allow performing studies that integrate better genomic and phenomic variations, increasing research translationality and reproducibility. Last but not least, it ensures transparency of previous studies while maximizing the utility of existing datasets. Several public resources have been set in place to access genomic data. 1000 Genomes Project [74], dbGaP [75], European Genome-phenome Archive [76] or the NHGRI AnVIL [77], a US cloud environment for the analysis of large genomic and related datasets, are some examples of databases that have provided services for the archiving and distribution of genetic and phenotypic data resulting from biomedical research projects during this pandemic.

Big data genomic and phenomic databases are necessary to promote personalized medicine but imply certain risks and challenges that need to be taken into consideration that can be roughly grouped into three main categories: issues associated with privacy, the occurrence of incidental findings and challenges associated with the safe management and sharing of genomic data. Researchers need to ensure that patients are well-informed about the benefits and potential risks of data sharing while educating participants about the importance of sample donation as the main pillar of scientific and medical progress. A great example is depicted in some sentences included in the 1000 Genomes Project consent template: "*there may be new ways of linking information back to you that we cannot foresee now. [...] We believe that the benefits of learning more about human genetic variation and how it relates to health and disease outweigh the current and potential future risks, but this is something that you must judge for yourself.*" Strategies to ensure patient privacy go from oversampling (recruiting more individuals than the final number to be included) to not collecting personal data besides sex. Aggregating data, such as allele frequency or allele-presence information, is another strategy that allows protecting participant privacy and also simplifies data sharing and storage. Genomic and phenomic databases usually incorporate controlled-access mechanisms to protect the privacy and confidentiality of research participants, limiting and/or restricting access to personal information. Implement technology safeguards to prevent unauthorized access, use, or disclosure of confidential and private data is a common feature of most of them. Data at EGA, for example, is collected from individuals that authorize data release only for specific research use, and strict protocols govern how information is managed, stored and distributed. EGA databases contain several measures to ensure the security of data, including a regular risk assessment and mitigation, audit logs, cryptography and communication security, among others [76]. IT security has become especially relevant with the transition from locked filing cabinets to digital databases, which bring enormous opportunities for big data analysis but also have an additional set of risks that need to be taken into account. NHGRI AnVIL, for example, uses NIST 800-53 Rev 4 security controls at the Moderate baseline and NIST 800-53 privacy controls documented, security protocols similar to those established in the industry [77].

The COVID-19 Host Genetics Initiative represents a good example of a huge worldwide effort made for COVID-19 genetic and phenotypic data sharing (+160 registered studies +19 countries). This initiative allows submitting individual-level data that includes genetic and clinical phenotype data and also study summary statistics. Individual-level data is shared via the European Genome-phenome Archive (EGA) or via NHGRI AnVIL (US studies). Researchers are allowed to download the metanalysis summary statistics as they are released by the consortia and also have a genome browser that allows them to explore all genetic variations found associated with infection and/or disease severity. Researchers can also apply for study-specific summary statistics and can also request access to the initiative’s data deposited on EGA and AnVIL via their respective Data Access Committee, which is composed of the PIs of the studies that have deposited the data (EGA) or managed directly by the NIH (AnVIL). Several genetic studies from Spain, including some launched from Andalusia, are currently contributing with data and samples to this initiative, which is currently working together with the EGA and with the ELIXIR network to establish the EGA Federation network and ensure that data from all countries can be deposited within all national jurisdictions [78]. 

## 7. Translating Personalized Medicine into Clinical Practice: The Andalusian Experience 

Andalusia, located in the south of Spain and with 8.5 million inhabitants, is the third most populated region in Europe, and it is larger than half of the countries of the European Union. Remarkably, Andalusia has the whole population under a unique universal electronic health record, thus forming the largest resource of this kind in the European Union. Under this scenario, all the decisions and strategies taken around this huge clinical database acquire enormous relevance. Since 2001, the data recorded by the Andalusian Public Health System (SSPA) are systematically uploaded to the Population Health Base (BPS), making it one of the largest repositories of highly detailed clinical data in the world (with over 13 million registries) [79]. BPS constitutes a unique and privileged environment to carry out large-scale RWE studies. Actually, one of the BPS missions is facilitating the discovery of new biomedical knowledge by means secondary use of clinical data [80], paying special attention to the evaluation of impact in personal data protection [81].

The Andalusian SARS-CoV-2 genomic surveillance project [82] set the ground for the implementation of a clinical circuit for controlling the COVID-19 pandemic as well as other potential future emergent viruses. This project engaged the 16 main tertiary hospitals in Andalusia, along with three research centers with genome sequencing facilities (IBIS, Genyo and CABIMER) and the Bioinformatics Area of the Progress and Health Foundation in a circuit of genomic data production. In parallel, the COVID-19 registries from Public Health and the BPS provide ongoing and retrospective clinical data, respectively, to the Bioinformatics Area, where the data are linked to the genomic data in a circuit of genomic data interpretation. Bioinformatics then provide (i) to the microbiologists at hospitals with information on the lineage, clade and relevant mutations in the virus; (ii) to Public Health with epidemiological data; (iii) to BPS with the viral sequences for further secondary clinical data studies; (iv) to the research community with the viral genome sequences through the European Nucleotide Archive (ENA).

The Andalusian Health Research and Innovation Strategy 2020–2023, presented on 2 September 2020, focused on the improvement of the wellbeing of citizens in the framework of Horizon Europe 2027, including a response to the impact of the challenges of the SARS-CoV2 pandemic. The Regional Health Ministry has been working and collaborating on different initiatives for some time: (a) To promote Digital Clinical records (Diraya®), integrating all the information on people into a Single Health Record and facilitating access to all the services and provisions of the health system, ensuring that all the relevant information is structured. As opposed to systems that merely assemble records, the design of the applications in Diraya shared tables, codes and catalogues; (b) To create the Bioinformatics Research Area [82], on 14 June 2016, to improve the technological support of personalized medicine, genomics and clinical genetics programs in the SSPA; (c) To create, by resolution of the management direction of the Andalusian Health Service 11 March 2018, the information system called BPS of the Andalusian Public Health System, which integrates clinical and epidemiological data from each patient; (d) The resolution of 9 March 2018, of the Public Business Entity Red.es, by which the Agreement with the Andalusian Health Service is published for the application of Information and Communication Technologies in the management of chronicity and continuity of care in the SSPA; (e) On 27 June 2019, at the meeting of the board of the Progress and Health Foundation (FPS), the creation of the B-D Area in Health of Andalusia was approved as an area integrated into the FPS in order to provide the SSPA of a platform of powerful, safe and data analysis tools oriented to health results and the optimization of healthcare processes based on personalized medicine; (f) During the last quarter of 2020, the coordination of the information and communication technologies (ICT) strategy of the SSPA was promoted; (g) The promotion of research on COVID19 in Andalusia, as of December 6, the number of research studies related to COVID-19, presented and/or evaluated in our Research Ethics Committees in Andalusia has been 277, with participation in 24 clinical trials addressing all the spectrum of COVID-19 from epidemiology, diagnosis, biomarkers, genetics (as a contributing study of the COVID-HGI), therapeutic interventions and vaccination. Some of them granted under the specific support for financing research, development and innovation (R+D+i) in COVID-19; (h) The Ministry of Health and Families, through its General Secretariat for Research, Development and Innovation in Health, has also set up three working groups: (1) prospective studies on the evolution of the pandemic; (2) personalized medicine in Covid-19; and (3) supplement and nutritional intervention against the SARS-Cov2 virus.

**Figure 1:**
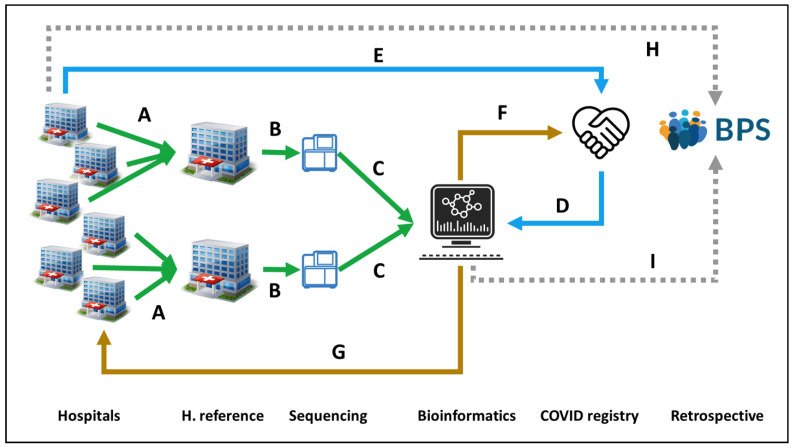
Circuit for COVID19 genomic surveillance. (**A**) Two reference Hospitals, San Cecilio and Virgen del Rocio, collect SARS-CoV-2 samples from Eastern and Western Andalusia, respectively. (**B**) Samples are sent to GENYO or IBIS sequencing facilities. (**C**) Sequencing data are sent to the Bioinformatics Area for processing and (**D**) linking to metadata from the COVID registry, (**E**) previously collected from the Hospitals. (**F**) Bioinformatics reports relevant epidemiological information to the COVID registry and (**G**) information on lineages and variants to the Hospitals for supporting clinical decisions. (**H**) Clinical data on COVID-19 patients recorded in the Hospitals is stored in the BPS. (**I**) Viral genomes are also stored in BPS linked to the rest of the patient’s clinical data for further secondary use.

Implementing personalized medicine in Covid19 included developing actions to define by means of BD and AI the interaction of genomics, epigenetics, metagenomics and viral sequencing in the development of events such as infection, severe disease, response to treatment and response to vaccination. A joint instruction was carried out on January 2020 from the General Secretariat for Research, Development and Innovation in Health and the Management Directorate of the Andalusian Health Service for the Management of samples in the approach to Personalized Medicine in COVID-19. Healthcare professionals will also have access to SARS-CoV-2 virus complete sequencing study by electronic biochemical request (MPA). The San Cecilio Clinical Hospital for Eastern Andalusia and Virgen del Rocio University Hospital for Western Andalusia were established as reference centers for receiving viral samples (Figure 1A) and sequencing them, respectively (Figure 1B), and the Bioinformatics Area process sequencing data (Figure 1C), joint with COVID registry metadata (Figure 1D), previously collected from the Hospitals (Figure 1E), and reporting back relevant epidemiological information to the COVID registry (Figure 1F) and information on lineages and variants to the Hospitals for supporting clinical decisions (Figure 1G). As previously stated, the clinical data of the Andalusian Health System is stored in the BPS (Figure 1H), but in this case, viral genomes are also stored in BPS (Figure 1I) linked to the rest of the patient’s clinical data, offering an unprecedented opportunity for large-scale secondary studies and implementation in clinical practice (Figure 1). Finally, the Bioinformatics Area submits the viral sequences to the ENA database, which is available to the scientific community. Since February, more than 2000 whole viral genomes have been sequenced, allowing the construction of a resource that depicts the evolution of SARS-CoV-2 along time and across the geography of Andalusia [82]. This systematic genomic surveillance system has allowed following the increase of the B.1.1.7 since February to become a majority or to detect new VOCs, such as the Brazilian lineage P1 or the South African B.1.351, and VOIs such as the Ugandan variant A.23.1 or others. 

## 8. Concluding Remarks

The pandemic has pushed us to a new scenario promoting association and relationship between governments and the scientific community at the same time that emerged multidisciplinary teams to take care of this complex disease together with telemedicine to guarantee health care keeping at home. Deep sequencing, bioinformatic area and clinicians working on personalized medicine could help to better understand the interaction between the virus and the host. These tools should be available for physicians able to include in their everyday decision-making process. The increasing need for personalized medicine supported by scientific and objective data, big data and AI systems to create algorithms based on individual variables (genomic), the host and the guest (pathogen and patient subject). Public and private investment for the generation and transfer of knowledge could support the development of high-quality translational and collaborative research to face a threatening situation similar to this terrible pandemic. 

## Figures and Tables

**Table 1:** List of SNPs significantly associated with COVID-19 risk of infection, hospitalization, or critical illness that have been identified by three of the major genetic studies that have analyzed host genetics. Chr: Chromosome. LD: linkage disequilibrium.

Chr	SNPs	Position	Genetic Variation(Effect Allele/Reference Allele)	Genes in LD Region	Associated Phenotype(s)	ß-Coefficient (COVID HGI) or ODDS RATIO (rest)	*p*-Value	Reference Study, nº of Patients & Phenotype(s) Definition
1	rs67579710	155203736	A/G	THBS3, KRTCAP2, TRIM46, MUC1, MTX1	Hospitalization	−0.138	3.4 × 10^−8^	*COVID HGI* *(Data freeze nº5 Jan 2021)* *46 studies across 19 countries worldwide* *Critically Ill (6.179)* *vs population control (1.483.780)* *Hospitalized COVID-19 (13.641)* *vs population control (2.070.709)* *SARS-CoV-2 infection (49.562)* *vs population control (1.770.206)* *PHENOTYPES:* *Critically ill: Required respiratory support or COVID-19 related death* *Hospitalized: Required hospitalization due to COVID-19* *SARS-CoV2 infection: * *Laboratory confirmed OR electronic health record, ICD coding OR Physician-confirmed COVID-19 OR self-reported COVID-19*
2	rs1381109	166061783	T/G	SCN1A	Hospitalization	−0.096	4.2 × 10^−8^	
3	rs10490770 rs11919389	45823240 101705614	C/T C/T	LZTFL1 RPL24, ZBTB11, CEP97, NXPE3	Critical Illness Hospitalization Infection susceptibility Infection susceptibility	0.634 0.5 0.149 −0.06	2.2 × 10^−61^ 1.4 × 10^−73^ 9.7 × 10^−30^ 3.5 × 10^−15^	
5	rs10070196	13939721	C/A	DNAH5	Infection susceptibility	0.044	9.7 × 10^−22^	
6	rs1886814	41534945	C/A	FOXP4	Hospitalization Infection susceptibility	0.233 0.101	1.1 × 10^−9^ 2.4 × 10^−8^	
8	rs72711165	124324323	C/T	TMEM65	Hospitalization	0.314	2.1 × 10^−9^	
9	rs912805253	133274084	T/C	ABO	Hospitalization Infection susceptibility	−0.103 −0.1	5.4 × 10^−10^ 1.5 X 10^−39^	
12	rs10774671	112919388	A/G	OAS1, OAS2, OAS3	Critical Illness Hospitalization Infection susceptibility	0.231 0.144 0.048	4.1 × 10^−13^ 6.1 × 10^−10^ 1.6 X 10^−11^	
17	rs1819040 rs77534576	46142465 49863303	A/T T/C	ARHGAP27, PLEKHM1, LINC02210 CRHR1, SPPL2C, MAPT, STH, KANSL1, LRRC37A, ARL17B, LRRC37AA2, ARL17A, NSF, WNT3 KAT7, TAC4	Hospitalization Critical illness	−0.129 0.369	1.8 × 10^−10^ 4.4 × 10^−9^	
19	rs2109069 rs74956615 rs4801778	4719431 10317045 48867352	A/G A/T T/G	DPP9 TYK2, ICAM1 ICAM3, ICAM4, ICAMS, ZGLP1, FDX2, RAVER1. PLEKHA4, PPP1R115A, TULP2, NUCB1	Critical Illness Hospitalization Infection susceptibility Hospitalization Critical Illness Infection susceptibility	0.231 0.144 0.048 0.36 0.236 −0.055	9.7 × 10^−22^ 2.8 × 10^−17^ 4.1 × 10^−9^ 5.1 × 10^−10^ 9.7 × 10^−22^ 1.2 × 10^−8^	
21	rs13050728	33242905	C/T	IFNAR2	Critical Illness Hospitalization	−0.20 −0.15	1.1 × 10^−16^ 2.7 × 10^−20^	
3	rs11385942	45876460	insertion–deletion GA or G	LZTFL1, SLC6A20, CCR9, FYCO1, CXCR6, XCR1	Severe Covid Intubation	1.77 (1.48–2.11) 1.70; (1.27 to 2.26)	1.2 × 10^−10^ 3.3 × 10^−4^	*COVID 19 * *Host(a)ge * *(1st release)* *(Spain+Italy)* *Severe Covid (1980) * *vs Population controls (2381)* *Severe Covid: * *Hospitalization + respiratory* *failure + confirmed SARS-CoV-2*
9	rs657152 rs8176719 rs41302905 rs8176747	Between 133255928 and 136139265	A/C 261delG A/G C/G	ABO	Severe Covid	A group 1.32 (1.20–1.47) O group 0.65 (0.53 to 0.79)	1.48 × 10^−4^ 1.1 × 10^−5^	
3	rs73064425	45901089	T/C	LZTFL1	Severe Covid	2.1 (1.88–2.45)	4.8 × 10^−30^	*GenOMICC * *(Genetics Of Mortality * *In Critical Care)* *UK* *Severe Covid (2771) * *vs Population control (45.875)* *Severe Covid:* *Patients in critical care, being profound hypoxaemic respiratory failure the archetypal presentation.*
6	rs9380142 rs143334143 rs3131294	29798794 31121426 32180146	A/G A/G G/A	HLA-G CCHCR1 NOTCH4	Severe Covid Severe Covid Severe Covid	1.3 (1.18–1.43) 1.9 (1.61–2.13) 1.5 (1.28–1.66)	3.2 × 10^−8^ 8.8 × 10^−18^ 2.8 × 10^−8^	
12	rs10735079	113380008	A/G	OAS1/3	Severe Covid	1.3 (1.18–1.42)	1.6 × 10^−8^	
19	rs2109069 rs74956615	4719443 10427721	A/G A/T	DPP9 TYK2	Severe Covid Severe Covid	1.4 (1.25–1.48) 1.6 (1.35–1.87)	4.0 × 10^−12^ 2.3 × 10^−8^	
21	rs2236757	33252612	A/G	IFNAR2	Severe Covid	1.3 (1.17–1.41)	2.3 × 10^−8^	

## Data Availability

Not applicable.
